# Are patients with chronic obstructive pulmonary disease at a greater risk for the development of autoimmune thyroiditis as an adverse event of immunotherapy in non-small cell lung cancer treatment?

**DOI:** 10.3389/pore.2025.1612022

**Published:** 2025-03-14

**Authors:** Andrej Zecevic, Ana Blanka-Protic, Aleksandar Jandric, Tatjana Adzic-Vukicevic

**Affiliations:** ^1^ Clinic for Pulmonology, University Clinical Center of Serbia, Belgrade, Serbia; ^2^ Faculty of Medicine, University of Belgrade, Belgrade, Serbia

**Keywords:** chronic obstructive lung disease, non-small cell lung cancer, immunotherapy, thyroiditis, adverse event

## Abstract

**Introduction:**

Immunotherapy has made a significant improvement in the treatment of patients with non-small cell lung cancer (NSCLC). It has a role in boosting the immune system, so it can fight cancer cells. Sometimes, this mechanism can lead to an overstimulation or misdirection of immune response, so it can act against the body itself. One of the organs most affected by this reaction is the thyroid gland, and there is no definitive explanation of the causes of this adverse event.

**Material and methods:**

In this retrospective observational study, we enrolled 103 patients with NSCLC and high PD-L1 expression (>= 50%) who were treated in our Clinic for pulmonology, University Clinical Center of Serbia, using Pembrolizumab as the first-line therapy.

**Results:**

Data analysis showed that 41 (39.81%) of 103 patients in our study had an adverse event of immunotherapy, and 21 of them had autoimmune thyroiditis (20.39%). Of all the patients, 19 of them were treated for chronic obstructive pulmonary disease (COPD) before the onset of Pembrolizumab. During treatment, eight of these patients developed thyroid dysfunction. Patients with COPD were at increased risk of developing autoimmune thyroiditis compared to non-COPD patients (OR 3.9 95% CI 1.135–13.260, p = 0.0227).

**Conclusion:**

Our study showed that patients dealing with COPD have a 3.9 times greater risk of developing autoimmune thyroiditis as an adverse event during Pembrolizumab treatment compared with patients without COPD.

## Introduction

Chronic obstructive pulmonary disease (COPD) and lung cancer are heavily related diseases [1]. They are both linked to cigarette smoking, as it leads to increased oxidative stress and inflammation in the airway, thus resulting in deoxyribonucleic acid (DNA) damage and impaired DNA repair [2]. Also, genetics and environmental factors (e.g., air pollution) play a tremendous role in both COPD and lung cancer pathogenesis [3].

Immunotherapy (IT) has made a significant improvement in the treatment of patients with NSCLC [4]. It has a role in boosting the immune system, so it can fight cancer cells.

IT’s main mechanism of action is the inhibition of immune checkpoints [5]. These checkpoints can be found on immune cells' surfaces, and they help these cells to recognize “good” from “bad” cells. The most known such checkpoint is programmed cell death protein 1 (PD-1) on T cells, and its interaction with programmed death-ligand 1 (PD-L1), which can be found on tumor cells, prevents immune cells from attacking tumor ones [6]. Some of IT affect ligands expressed on tumor cells while others PD-1 on immune cells.

Pembrolizumab is still the only IT used in Serbia for NSCLC treatment, as the first-line single therapy for patients with high (≥50%) PD-L1 expression in the IV stage of the disease, with negative driver mutations [7]. It works as an immune checkpoint inhibitor (ICI), inhibiting PD-L1 [5].

The aim of this study is to investigate the incidence and risk of developing autoimmune thyroiditis in NSCLC patients receiving immunotherapy, specifically comparing the odds of occurrence between patients with COPD and those without.

## Materials and methods

A retrospective observational study was conducted in a 3-year period (2021–2023.) with 103 patients with NSCLC and high PD-L1 expression (≥50%), who were treated in the Clinic for Pulmonology, University Clinical Center of Serbia, Belgrade, with Pembrolizumab, as the first-line therapy. In this study, the diagnosis of COPD in all patients was established using spirometry prior to the initiation of immunotherapy. The analysis was performed using the statistical program IBM SPSS version 27.

For analysis of categorical data Chi-square test was used, and for data with numerical variables, depending on the normality of the distribution, the Student t-test or Mann-Whitney test. All statistical methods were considered significant for the chosen level of confidence of 0.05.

## Results

The majority of patients in our study were men (62.14%), and the average age at the time of the diagnosis was 63.98 (±8.63 years). Demographic data are presented in Table 1.

**TABLE 1:** Patients’ baseline characteristics.

Patient characteristics	Ratio
Total Male Female	103 64 (62.14%) 39 (37.86%)
Age	63.98 (±8.63 years)
Tumor type Adenocarcinoma Squamocellcarcinoma NOS	71 (68.93%) 25 (24.27%) 7 (6.8%)
Average PDL-1 expression	75.69
Bronchoscopy FNAB other	56 (54.37%) 30 (29.13%)/17 (16.5%)
Smoking status active former non-smoker	72 (69.9%) 25 (24.27%) 6 (5.83%)
Comorbidities Yes No	74 (71.84%) 29 (28.16%)
COPD Yes No	19 (18.45%) 84 (81.55%)
Diabetes mellitus Yes No	15 (14.56%) 88 (85.44%)
Hypertension Yes No	40 (38.83%) 63 (61.17%)
Arrhythmias Yes No	12 (11.65%) 91 (88.35%)
Cardiomyopathy Yes No	8 (7.77%) 95 (92.23%)
Lethal outcome Yes No	12 (11.65%) 91 (88.35%)

Abbreviations: NOS (non-otherwise specified); FNAB (fine needle aspiration biopsy).

All patients had the IV stage of the disease, with the contralateral lung being the most common site of metastasis (49.51%). Other sites affected were the adrenal gland (20.39%), central nervous system (19.42%), pleura (16.5%), and bones (12.62%).

Every patient included in this study was treated with at least eight cycles of Pembrolizumab (the average number of cycles was 26). Twelve patients died during the period of observation (11.65%).

Of all the patients, 40 of them (38.83%) were treated for hypertension before the onset of Pembrolizumab. The second most common comorbidity was COPD in 19 cases (18.45%). Less frequent comorbidities were diabetes mellitus (14.56%), arrhythmias (11.65%), and cardiomyopathy (7.77%). Other malignancies (breast and laryngeal carcinomas) were present in 4.85% of all the patients.

During treatment, 41 out of 103 patients had immune-related adverse events (irAEs). The most common was autoimmune thyroiditis in 21 cases (20.39%). Other important side effects were dermatitis (9.71%), pruritus (also 9.71%), colitis (4.85%), and hepatitis (2.91%).

Data analysis showed the absence of significance between the duration of ICI treatment and occurrences of any irAEs (p > 0.05). Also, there wasn’t any association between comorbidities and irAEs incidence, except between COPD and autoimmune thyroiditis.

During the treatment period, eight patients with COPD developed thyroid hypofunction [8]. In the group of patients without COPD this incidence was lower (13/84) (Table 2). Patients with COPD were at increased risk of developing autoimmune thyroiditis compared to non-COPD patients (OR 3.9 95% CI 1.135–13.260, p = 0.0227).

**TABLE 2:** Comparative analysis of COPD and autoimmune thyroiditis in the study population.

	Autoimmune thyroiditis	Without autoimmune thyroiditis
With COPD	8	11
Without COPD	13	71

Only three patients had a transient thyrotoxic phase, and the other 18 developed hypothyroidism, requiring hormonal substitution therapy. All eight patients with COPD and thyroid hypofunction developed permanent hypothyroidism.

## Discussion

As we have already mentioned, there is a strong connection between COPD and lung cancer [1]. Between 40% and 70% of patients with lung cancer also have COPD [9], which is at least two times higher than in our study (less than 19%).

Generally, IT leads to less severe side effects of chemotherapy, as they are less toxic medications [10]. But, sometimes this mechanism of work can lead to an overstimulation or misdirection of immune response, so it can act against the body itself. This leads to irAEs that can go from mild to severe immune-related damage to different organs [11]. The most commonly found toxicities are hepatitis (2%–9%), diarrhea and colitis (1%–15%), thyroid dysfunction (up to 11%), and pneumonitis (3%–9%) [12].

One of the most affected organs by this reaction is the thyroid gland [13]. Lung cancer patients going through the treatment with ICI commonly experience thyroid dysfunction, which typically occurs in the early stages of the treatment. Only around 10% of these patients require some kind of intervention, such as delay of immunotherapy until the symptoms improve, levothyroxine for manifest hypothyroidism, and for patients with destructive thyroiditis, the initial thyrotoxic phase should be treated using β-blockers or a short course of high doses of glucocorticoids [14, 15].

There are a few theories about the mechanism of action that leads to these irAEs, such as the higher expression of PD-L1 and PD-L2 on the surface of thyroid cells [16], or thyroiditis mediated by CD56^+^CD16^+^ natural killer (NK) cells [17]. ICI induces the destruction of thyroid tissue (asymptomatic thyrotoxic phase), which consequently leads to the development of hypothyroidism, which is likely permanent [18]. Symptoms of thyroid hypofunction are fatigue, weight gain, and heart rate changes [19]. Some authors revealed that subsequent thyroid dysfunction in patients on IT is associated with a good prognosis [8].

Diagnosis of hypothyroidism is established on clinical anamnesis, examination, blood tests, and lastly thyroid ultrasound [19]. Ultrasound in this group of patients can detect localized or diffuse thyroiditis, suggesting an autoimmune disorder [15].

Well known connection between autoimmune thyroiditis and COPD was published before [20]. Previously literature observed that patients in earlier stages of COPD had a higher risk of developing thyroiditis [21]. A possible link between these two disorders is systemic inflammation, but also hypoxia and glucocorticoid use in treatment [22]. This is gravely important because hypothyreosis increases exacerbation risk in patients with COPD, by reducing respiratory muscle function and exercise capacity, leading to inspiratory and expiratory weakness in this group of patients [23]. The prevalence of autoimmune thyroiditis is significantly higher in higher stages of COPD (GOLD E) [24].

In our study, 20.39% of patients had thyroid dysfunction, which is almost two times more than in the literature [12]. All of them were referred to an endocrinologist for further evaluation of thyroid dysfunction. The percentage of patients who developed hypothyroidism from the asymptomatic thyrotoxic phase (85.7%) was similar to the data in the literature. All of them received levothyroxine, and they remained on hormone replacement therapy on the last follow-up, suggesting permanent hypothyroidism [17]. Hypothyroidism, on the other hand, was developed in all eight patients with COPD and thyroid hypofunction irAEs.

There are a few strengths of this study. First, the diagnosis of COPD was established using spirometry, ensuring a standardized and objective approach. Additionally, the findings have direct clinical relevance, potentially guiding the management and monitoring of these patients.

However, there are also a few limitations to consider. The retrospective design of the study may lead to selection bias. The sample size is just over 100 patients, which might affect the generalizability of the results. Also, the follow-up duration may have been insufficient to observe long-term outcomes and late-onset autoimmune thyroiditis.

## Conclusion

Our study showed that patients dealing with COPD have a 3.9 times greater risk of developing autoimmune thyroiditis as an irAE during Pembrolizumab treatment compared with patients without COPD. Before ICI administration patients should be carefully observed with permanent control of thyroid status.

## Data Availability

The raw data supporting the conclusions of this article will be made available by the authors, without undue reservation.
